# Mapping of a Subgingival Dual-Species Biofilm Model Using Confocal Raman Microscopy

**DOI:** 10.3389/fmicb.2021.729720

**Published:** 2021-10-05

**Authors:** Lukas Simon Kriem, Kevin Wright, Renzo Alberto Ccahuana-Vasquez, Steffen Rupp

**Affiliations:** ^1^Fraunhofer Institute for Interfacial Engineering and Biotechnology, Stuttgart, Germany; ^2^Procter & Gamble, Reading, United Kingdom; ^3^Procter & Gamble, Kronberg, Germany

**Keywords:** confocal Raman microscopy, biofilms, bacteria, subgingival, mapping, cluster analysis

## Abstract

Techniques for continuously monitoring the formation of subgingival biofilm, in relation to the determination of species and their accumulation over time in gingivitis and periodontitis, are limited. In recent years, advancements in the field of optical spectroscopic techniques have provided an alternative for analyzing three-dimensional microbiological structures, replacing the traditional destructive or biofilm staining techniques. In this work, we have demonstrated that the use of confocal Raman spectroscopy coupled with multivariate analysis provides an approach to spatially differentiate bacteria in an *in vitro* model simulating a subgingival dual-species biofilm. The present study establishes a workflow to evaluate and differentiate bacterial species in a dual-species *in vitro* biofilm model, using confocal Raman microscopy (CRM). Biofilm models of *Actinomyces denticolens* and *Streptococcus oralis* were cultured using the “Zürich *in vitro* model” and were analyzed using CRM. Cluster analysis was used to spatially differentiate and map the biofilm model over a specified area. To confirm the clustering of species in the cultured biofilm, confocal laser scanning microscopy (CLSM) was coupled with fluorescent *in vitro* hybridization (FISH). Additionally, dense bacteria interface area (DBIA) samples, as an imitation of the clusters in a biofilm, were used to test the developed multivariate differentiation model. This confirmed model was successfully used to differentiate species in a dual-species biofilm and is comparable to morphology. The results show that the developed workflow was able to identify main clusters of bacteria based on spectral “fingerprint region” information from CRM. Using this workflow, we have demonstrated that CRM can spatially analyze two-species *in vitro* biofilms, therefore providing an alternative technique to map oral multi-species biofilm models.

## Introduction

Microorganisms tend to attach, assemble, proliferate, and form clusters on surfaces enabling their survival under many different conditions and are commonly referred to as biofilms. Of the many varieties studied, oral biofilms have been studied in relation to oral diseases, like gingivitis and periodontitis (Tanner et al., 1996; Abusleme et al., 2013). Subgingival biofilms are specifically important because they grow in the subgingival sulcus and are in direct contact with both the tooth surface and tissue cells, where bacteria accumulation could lead to gingivitis or the development of other periodontal infections. Key subgingival microorganisms were identified previously by Socransky et al. (1998) and were further specified by Abusleme et al. (2013). In general, two of these genera, *Actinomyces* and *Streptococci*, are two major groups found in the subgingival biofilms of both healthy (52.8%) and infected individuals (43.9%; Ximenez-Fyvie et al., 2000). It has been shown that these microorganisms tend to form clusters in subgingival biofilms, with dense “hotspot” areas composed of one of these species (Guggenheim et al., 2009; Zijnge et al., 2012).

While the microbial composition of biofilms plays a key role in diseases, the architecture of the biofilm has an important role in contributing to the understanding of the disease mechanism. *Streptococci* sp. and *Actinomyces* sp. are both considered early or initial colonizers, attaching primarily to surfaces, while *Actinomyces* sp. also attach to other early colonizing microorganisms like *Streptococci* sp. (Kolenbrander and London, 1993). Common techniques used in understanding biofilm architecture include scanning electron microscopy (SEM), atomic force microscopy (AFM), transmission electron microscopy (TEM), and confocal laser scanning microscopy (CLSM). To date, fluorescent *in situ* hybridization (FISH) coupled with CLSM is considered the state-of-the-art technique for oral biofilm architecture analysis because it allows for the analysis of architecture, while at the same time also considering species differentiation (Kommerein et al., 2017; Xiao et al., 2017; Thurnheer et al., 2019). Even though these techniques produce high-resolution results and allow for the characterization of the species within a biofilm, they can cause destructive sampling and staining effects, compromising the samples integrity, therefore highlighting the current lack of a simple, non-destructive, and cheap preparation procedure (Pantanella et al., 2013).

In recent years, optical spectroscopy techniques have demonstrated technical advancements in identifying specific chemical components with high spectral resolution. One of these techniques, confocal Raman microscopy (CRM), measures scattered radiation and energy shifts from samples after they were excited with a laser beam. Previously, we demonstrated the use of CRM to identify different oral microbes in pure culture (Kriem et al., 2020). Key advantages of this technique beyond microbial identification are: (1) very minimal sample preparation, (2) affordable measurements, and (3) the opportunity of continuous evaluation of the same location due to the technology’s non-destructive nature of measuring a sample. In contrast to CLSM, the CRM Raman technique is able to identify microorganisms without causing staining and destruction effects, consequently providing the opportunity for more information to be gathered.

Previous studies successfully demonstrated that CRM is capable of spatially differentiating complex biomedical samples such as tissues and bacteria (Strola et al., 2013; Gualerzi et al., 2017; Rebrošová et al., 2017; Sil et al., 2017; Cals et al., 2018). To the best of our knowledge, CRM has, so far, only been used a few times for the analysis of environmental biofilms. The focus of these studies were based on molecular details of spectra: (1) of single species biofilms (Kusić et al., 2015; Ramirez-Mora et al., 2019; Gieroba et al., 2020; Wickramasinghe et al., 2020), (2) over time (Chao and Zhang, 2012; Carey et al., 2017; Keleştemur et al., 2020; Liu et al., 2020), and (3) under the influence of stress, mostly chemicals (Jung et al., 2014; Daood et al., 2020). All in all, these studies lacked a spatial analysis when using Raman spectra.

Scientific advances have also been made in the use of surface-enhanced Raman scattering (SERS) for the analysis of biofilms, allowing higher levels of discriminating spectra (Chao and Zhang, 2012; Keleştemur et al., 2018, 2020). However, this analysis focuses on chemically un-modified biofilm samples and surfaces, and for that reason, SERS is not considered in this study.

Bacteria in subgingival biofilms occupy the same habitat and, therefore, show similar chemical compositions. We previously demonstrated that differentiation between species can be successfully achieved using spectral fingerprint patterns and multivariate statistical models for five subgingival species from four different “Socransky complexes” (Socransky et al., 1998; Kriem et al., 2020). Additionally, we successfully determined and differentiated mono-species biofilms based on their spectral fingerprint region. In this process, partial least square (PLS) was used to differentiate each species.

Principle component analysis (PCA) is another powerful analysis tool that was used in the previous studies (Lu et al., 2011; Almarashi et al., 2012; Jung et al., 2014; Colniță et al., 2017; Gualerzi et al., 2017; Sil et al., 2017). Cluster analysis (CA) was also used successfully in previous studies, but with different applications (Parthasarathy et al., 2008; Bonifacio et al., 2010; Hutchings et al., 2010; Maitra et al., 2020). For these reasons, PCA was selected as the multivariate tool to demonstrate the differentiation of the species, and CA was selected to map the two-species biofilms in this research, because CA allows the direct comparison of centroid spectra of the formed clusters with calibration spectra.

In the past, a limited number of studies used chemometric information for the spatial distribution of bacteria in biofilms. Beier et al. (2010) were able to demonstrate that it is possible to differentiate two *Streptococci* sp. in a pseudo-biofilm sample (sample with the two species mixed artificially to create the appearance of a cultured biofilm) and were able to confirm differentiation of species by PC-LR. This analysis, however, was based on modified bacteria by staining and uncultured biofilms over time. Gieroba et al. (2020) were able to use chemical maps to locate glucans and Amide I for different *Streptococci* sp. While they were able to determine concentrations of different chemical compounds in a specified area, the research’s focus was not on the differentiation of species in a multi-species biofilms based on their chemometric spectra and lacked the statistical evaluation that resulted in the chemical maps. Horiue et al. (2020) were able to map bacterial species in pink biofilms using specific pigment spectral bands and statistical methods. While it was possible to differentiate the areas, the study was unable to associate clusters to specific bacterial species.

In this research, we hypothesize that Raman spectroscopy in combination with multivariate analysis techniques, such as a PCA and CA, would be able to predict and differentiate two subgingival bacteria in a two-species biofilm model. To develop and confirm this approach, we used different datasets, samples, and analysis technologies to confirm: (1) species clustering in the *in vitro* grown subgingival two-species biofilm model, (2) the use of a multivariate analysis model in a mapping setup, and (3) the use of chemometric information from Raman spectra for the mapping of *in vitro* two-species biofilm models. The purpose of this study was to investigate the use of CRM as an alternative technique for the *in vitro* research of subgingival biofilm models using a stepped approach. In the first step, biofilm clustering was evaluated using CLSM. Distribution analysis using CRM and a multivariate analysis was then performed on dense bacteria interface area (DBIA) samples using planktonic bacteria. These results were compared to morphology and CLSM. Lastly, artificially grown two-species biofilms were mapped using the previously developed workflow and compared to morphology measurements of the biofilm.

## Materials and Methods

### Planktonic and DBIA Sample Preparation

Laboratory stocks of *Streptococcus oralis* (DSM20066) and *Actinomyces denticolens* (DSM20671) were cultured in Falcon tubes with Brain Heart Infusion Medium (Sigma-Aldrich) and incubated under anaerobic conditions (80% N_2_, 15% CO_2_, and 5% H_2_) at 37°C for 24h. Afterward, each bacterial stock was diluted in five new tubes with fresh modified fluid universal medium (mFUM) according to Gmür and Guggenheim (1983) for a total of 96h. This sample preparation procedure was repeated for a total of three times per species.

For calibration spectra, the sample was put in Eppendorf tubes and centrifuged at 5,000rpm for 5min. After centrifugation, the supernatant was removed. Samples were then resuspended in DI water and centrifuged for an additional 5min at 5,000rpm. After centrifugation, the supernatant was removed, and the bacterial pellet was vortexed for 15s in order to suspend the pellet. A total of 3μl was dispensed on a borosilicate glass slide (VWR) and air-dried for 10min for confocal Raman spectral analysis. For the calibration spectra, a total of 15 spots were used for spectral acquisitions.

Dense bacteria interface area samples were generated by dispensing 3μl of *A. denticolens* on a glass slide, air-drying for 10min, then dispensing 3μl of *S. oralis* next to the *A. denticolens* drop and drying it for an additional 10min to receive a defined interface between the two species.

### Biofilm Sample Preparation

Glass coupons of 1cm^2^ were cut from borosilicate glass slides (VWR) and sterilized. Mono- and dual-species biofilms were grown on coupons in 24-well culture plates (VWR) using similar materials and methods for biofilm formation presented elsewhere (Guggenheim et al., 2001). In short, wells with glass coupons were filled with a mixture of PBS at pH 7.2 (800μl), and mFUM (800μl), and had a final glucose concentration of 0.15% (w/v; Gmür and Guggenheim, 1983; Guggenheim et al., 2001). For mono-species biofilms, wells were inoculated with one bacterial species adjusted to 1.0 OD_600_ (200μl). Additionally, for multi-species biofilms, the bacteria were adjusted to 1.0 OD_600_ and mixed at 1:1 ratio for a total volume of 200μl. Biofilms were cultivated under the same conditions as described in planktonic and DBIA sample preparation. Biofilm coupons were removed from the wells after 65h, dip-washed in DI water three times, and placed on glass slides (VWR) to be dried at room temperature for 10min. In total, 15*S. oralis* mono-species biofilms, 15 *A. denticolens* mono-species biofilms, and 15 dual-species biofilms were cultivated.

### Structural Analysis of Biofilms

The structural analysis and comparison to Raman microscopy was done using FISH. The staining was performed according to the protocol previously described by Thurnheer et al. (2004) using the probe combinations listed in Table 1. In short, biofilms were fixed using 4% paraformaldehyde. After fixation, biofilm samples were pre-hybridized in hybridization buffer [0.9molL^−1^ NaCl, 20mmolL^−1^ Tris–HCl, (pH 7.5), 0.01% sodium dodecyl sulfate, and 30% formamide] at 46°C for 15min without oligonucleotide probes. Then, biofilms were hybridized for 90min with specific oligonucleotide probes at the same temperature. Samples were washed in washing buffer [102mmolL^−1^ NaCl, 20mmolL^−1^ Tris–HCl (pH 7.5), 5mmolL^−1^ EDTA, and 0.01% sodium dodecyl sulfate] for 45min at 48°C. For CLSM analysis, the samples were washed with 0.9molL^−1^ NaCl, and then fixed onto glass slides and embedded in Mowiol® 4-88 (Sigma-Aldrich).

**Table 1:** Sequence and formamide concentrations for fluorescent *in situ* hybridization (FISH) probes.

Organism	Name	Label	FA^a^ (%)	NaCl^b^ (mM)	Probe concentration (μg/ml of hybridization buffer)	Sequence (5'→3')	Source
*Streptococcus oralis*	MIT447	6-FAM	30	102	20	CAC CCG TTC TTC TCT TAC A	Thurnheer et al., 2004
*Actinomyces denticolens*	ACT476	Cy3	30	102	20	ATC CAG CTA CCG TCA ACC	Gmür and Lüthi-Schaller, 2007

a Formamide concentration in the hybridization buffer.

b Concentration of NaCl used in the washing buffer.

To analyze the architecture of the grown biofilms, we used CLSM. In total, 15 fluorescent labeled coupons were analyzed using an inverted microscope Zeiss LSM710 (Zeiss, Oberkochen, Germany) fitted with an Axio Observer Z1, a UV laser, an Ar laser (Lasos, Jena, Germany), a He-Ne laser (Zeiss, Oberkochen, Germany), and a computer-operated confocal laser scanning system. Filters were set to 500–540nm for detection of 6-FAM (green color on image) and 570–630nm for Cy3 (red color on image). Images were obtained with a 100× oil immersion objective. Each biofilm-attached coupon was scanned at a random position. Further, a Z-stack was generated with a slice thickness set to 1.018μm. Images were processed and recombined using ImageJ (Rasband, 1997).

Morphological images were acquired using a Renishaw inVia Qontor with a 100× objective. This allowed the acquisition of the same area for microscopic and Raman analysis. Due to the sample preparation procedure for FISH/CLSM and the location limitations, it was not possible to view the exact same area in FISH/CLSM as in microscopic and Raman analysis. However, samples were used from the same batch of experiments to ensure comparable biofilms.

### Raman Data Acquisition, Processing, and Microscopic Imaging

The instrument used for Raman analysis was a Renishaw inVia Qontor, which was equipped with a 100× objective. In a previous publication by the same authors, a ThermoFisher Scientific DXR2xi instrument was used for acquisition. Renishaw inVia Qontor was used in this research because of its increased specificity and signal count compared to the ThermoFisher Scientific DXR2xi, thus being able to show minor bands (Kriem et al., 2020). The instrument was equipped with a 532nm filter and a 532nm laser to capture a spectral range of 282.8–2016.2cm^−1^ and 1,015 collection points. Data acquisition was performed using 50% laser power (equivalent to 25mW), 1s exposure time and 10 accumulations. For every planktonic and biofilm sample, 20 random points were chosen in the image window on the bacteria-coated or biofilm glass slide for spectra to be taken. For mapping of DBIA samples and dual-species biofilms, a random window was selected. Acquisitions were taken over an 18μm×18μm area in 1μm steps for a total of 324 spectra. For dual-species biofilms, random locations that were not close to the sample border were chosen. For DBIA samples, an interface area between the two species was selected. For both sample acquisitions, microscopic images were taken for morphology analysis of the same area. The two species used for the confirmation of the model have different morphological shapes. As *A. denticolens* are rod-shaped with a size of 0.2–1.0μm by 2.0–5.0μm and *S. oralis* are spherical-, ovoid-, or cocci-shaped with a size of 0.5–2.0μm, the species can be distinguished based on their appearance and therefore allow for the use of morphology for differentiation in this work.

All spectral analyses and processing were performed using Renishaw WiRE 5.4 (Renishaw plc, Wotton-under-Edge, United Kingdom). Each spectrum was preprocessed the same way to reduce noise effects and spectral variations, due to spectral sample collection, and spectra with oversaturated signals were removed before processing. Firstly, the spectral range was reduced to the “fingerprint” area from 600 to 1,800 cm^−1^, baseline noise from the borosilicate background was deleted using cosmic ray removal and baseline subtraction using intelligent polynomial algorithms, and noise filtering was applied. To additionally reduce noise, a seven-point Savitzky–Golay smoothing was applied to the spectra. Finally, all spectra were normalized on a scale from 0 to 1 and differentiated by second-order Savitzky–Golay differentiation with a window size of 9 and second polynomial order.

### Multivariate Analysis and Mapping

**Figure 1:**
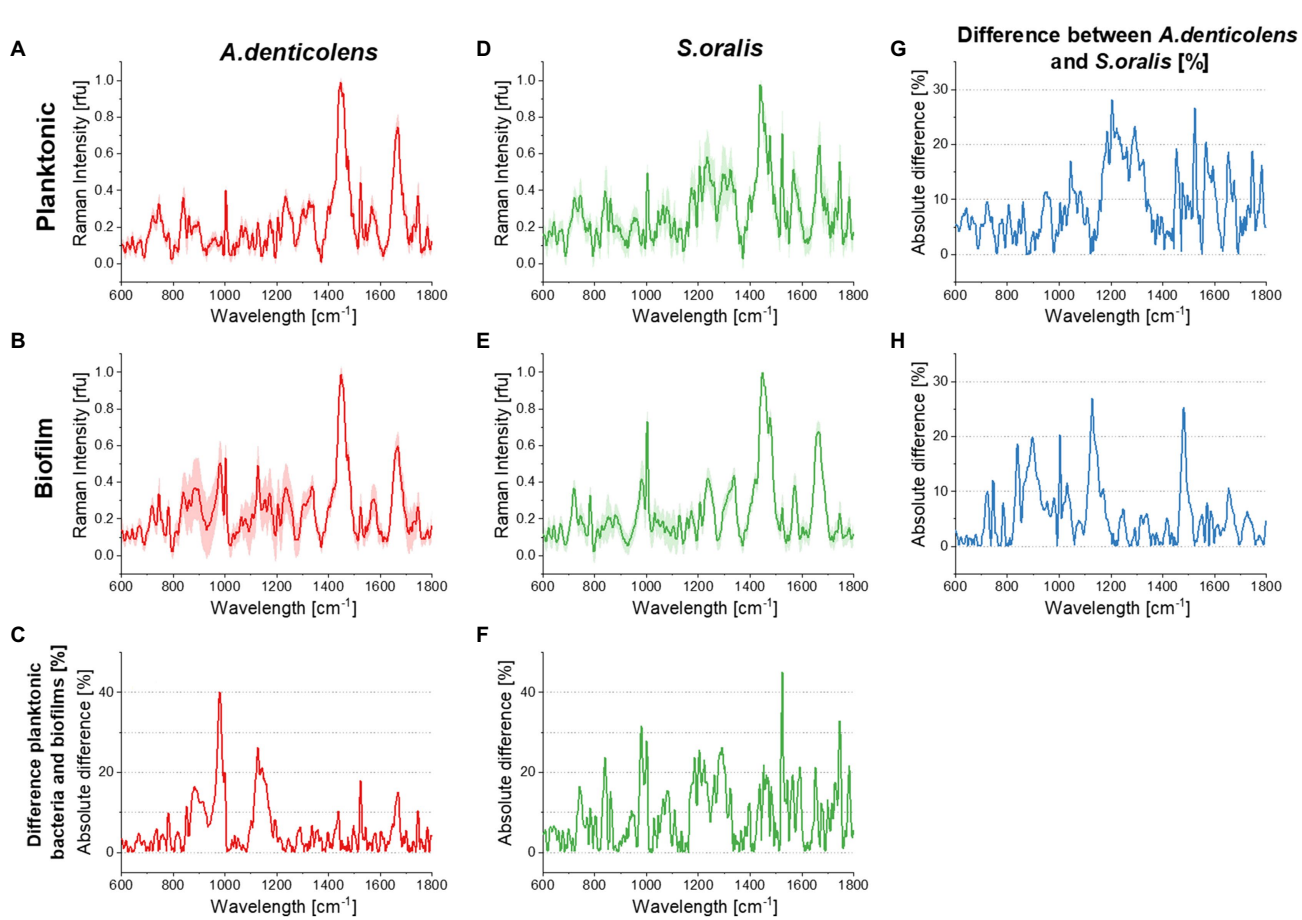
Averaged processed Raman signals (300 spectra for each dataset) before Savitzky–Golay differentiation for *Streptococcus oralis* and *Actinomyces denticolens* and their SDs. **(A)** shows the spectra for *A. denticolens* for planktonic bacteria and **(B)** for mono-species biofilms. **(C)** shows the absolute % difference between planktonic bacteria and mono-species biofilm spectra. **(D)** shows the *S. oralis* spectra for planktonic bacteria and **(E)** for mono-species biofilms. **(F)** shows the absolute % difference between planktonic bacteria and mono-species biofilm spectra. **(G)** shows the absolute % difference between *A. denticolens* and *S. oralis* for planktonic bacteria and **(H)** for mono-species biofilms.

Planktonic bacteria and mono-species biofilm calibration spectra were analyzed by PCA using Origin 2019b (OriginLab Corp., Northampton, MA, United States) considering the extraction of two components. Results were then shown with PC1 on the *X*-axis and PC2 on the *Y*-axis including a 95% confidence ellipse of the pre-defined two groups of *S. oralis* (green) and *A. denticolens* (red). PCs were assigned based on the collected spectra by using the mean of each species (shown in Figure 1) where PCs represent the eigenvectors corresponding to the calculated eigenvalues to capture the maximum variance of all the data points. The used two PCs in this work were the principal components that showed the highest variances.

After processing the mapping datasets for each of the biofilm samples, they were analyzed using CA integrated in WiRE 5.4. CA is used to group a defined set of spectra into clusters of similar spectral information. These clusters have a centroid spectrum, which is the mean of all the spectra in the clusters they are affiliated with. In this research, CA was performed using K++, fuzzy c-means with 2.0 fuzzification parameters, Euclidean distance metrics and 10 iterations and restarts. The centroid spectra were then compared to the calibration spectra generated from planktonic bacteria for DBIA samples, while mono-species biofilm calibration spectra were used for the analysis of dual-species biofilms. Depending on the cluster affiliation, each spectral point in the same group was colored accordingly (red for *A.denticolens* and green *S.oralis*) and mapped.

Microscopic image analysis based on morphology of *A. denticolens* and *S. oralis* was performed using morphological segmentation in the Plugin MorphoLibJ in ImageJ of the imaged area that was also analyzed by Raman. Here, images were analyzed based on morphological operations. First, images were converted into gray-level images. Based on the shape of structures (here individual bacterial shapes) as well as color, the structures were assigned accordingly. Coverage of the species for Raman mapping and morphology was evaluated using ImageJ (Rasband, 1997).

## Results

**Figure 2:**
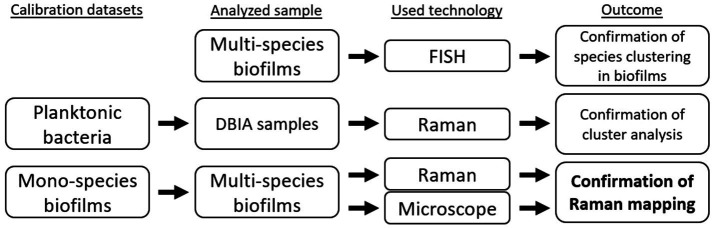
Experimental design setup for the differentiation and confirmation of Raman mapping in a two-species biofilm model.

In this work, we used different datasets, samples, and analysis technologies to confirm: (1) species clustering in the *in vitro* grown subgingival two-species biofilm model, (2) the use of a multivariate analysis model in a mapping setup, and (3) the use of chemometric information from Raman spectra for the mapping of *in vitro* two-species biofilm models. Figure 2 shows a schematic of the experimental designs used in the work to generate different outcomes.

### Confirmation of Biofilm Architecture

**Figure 3:**
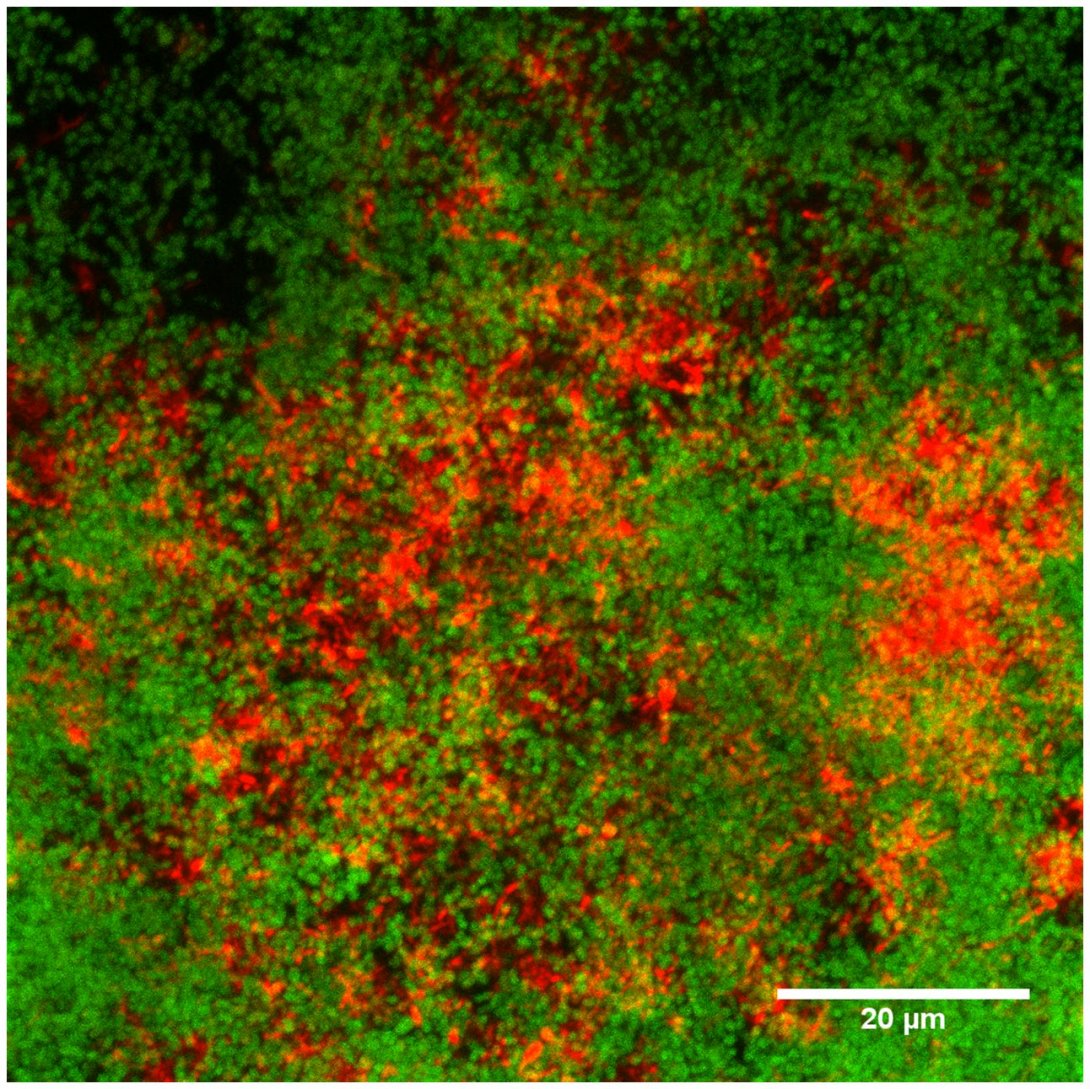
Confocal laser scanning microscopy (CLSM) image of the FISH-stained two-species biofilm. Sixteen individual images with a *z*-step size of 1.018μm of the biofilm stained with species-specific 16S rRNA FISH probes for *S.oralis* (MIT447, green) and *A.denticolens* (ACT476, red) were overlaid to one image using ImageJ. Individual images are available in Supplementary Material.

Biofilms were stained with two-species specific oligonucleotides (6-FAM for *S. oralis* and Cy3 for *A. denticolens*) and assessed by CLSM. The image shown in Figure 3 demonstrates that it was possible to simultaneously stain species by FISH with the described settings above, even though there was an overlap in excitation wavelengths for the two stains. *S. oralis* (green color) was more distributed in the biofilm than *A. denticolens* (red color) was. Distribution patterns in the biofilm also showed cluster formations in areas that were predominantly *S.oralis* and other areas that were predominantly clustered with *A. denticolens*. Figure 3 displays a combined image of 16 individual images from a *Z*-Stack (individual images are provided in Supplementary Material). Due to the layering of multiple images, some areas appear in a yellow color. However, these yellow colors originate from having both bacterial species present in the same spot in different layers and can mostly be observed in transition areas of the clusters.

### Calibration of Planktonic Bacteria and Mono-Species Biofilms

Spectra of *A. denticolens* and *S. oralis* were analyzed using CRM as described in Materials and Methods. Figure 1 shows the plots of averaged Raman spectra (300 spectra per strain and condition). Specific vibrational bands were assigned in a previous study completed by us (Kriem et al., 2020). Reference databases formerly demonstrated that it is possible to identify dominant chemical signature patterns that can be found and differentiated in Raman spectra (De Gelder et al., 2007; Kumar et al., 2016; Sil et al., 2017; Kriem et al., 2020). Major common bands in microbiological samples are Amide I and Amide III, which can be identified at ~1,250cm^−1^ (Amide III) and~1,660cm^−1^ (Amide I). Amino acids can be found at multiple wavelengths. In these studies, oral bacteria were mostly identified as phenylalanine are seen at ~1,000cm^−1^ and C-N and C-C stretches (specific for proteins) at ~1,125cm^−1^. Additionally, the presence of lipids could be seen as CH_2_ deformations at ~1,450cm^−1^. These described band patterns were found across both oral bacteria species in planktonic and biofilm conditions (Figure 1). *Actinomyces denticolens* only showed small band differences between planktonic and biofilm bacteria. The biggest differences appeared at ~982cm^−1^ (Polysaccharides) and ~1,129cm^−1^ (C-N, C-C stretch Protein; Figure 1C). *Streptococcus oralis* however showed a change of bands in multiple regions, most dominantly between 800–1,000cm^−1^ and 1,500–1,750cm^−1^ (Figure 1F). These differences however were not referred to the presence or absence of bands but mostly the height of these bands. This indicates an up- or downregulation of processes or cellular compounds within the cell due to its condition of being either in a planktonic or biofilm condition. This was also seen when comparing species. Most bands were present for both *A. denticolens* and *S. oralis*, but the heights of these bands differ. No pattern of differences can be identified for planktonic and biofilm bacteria. Figure 1G however shows that band differences between the two species appear, and for that reason, the model allows the use of planktonic bacteria for DBIA samples to validate the method for bacteria differentiation using Raman microscopy and biofilm spectra for dual-species biofilm analysis (Figure 1H).

### Establishing Reference Spectra for Planktonic Bacteria and Mono-Species Biofilms

**Figure 4:**
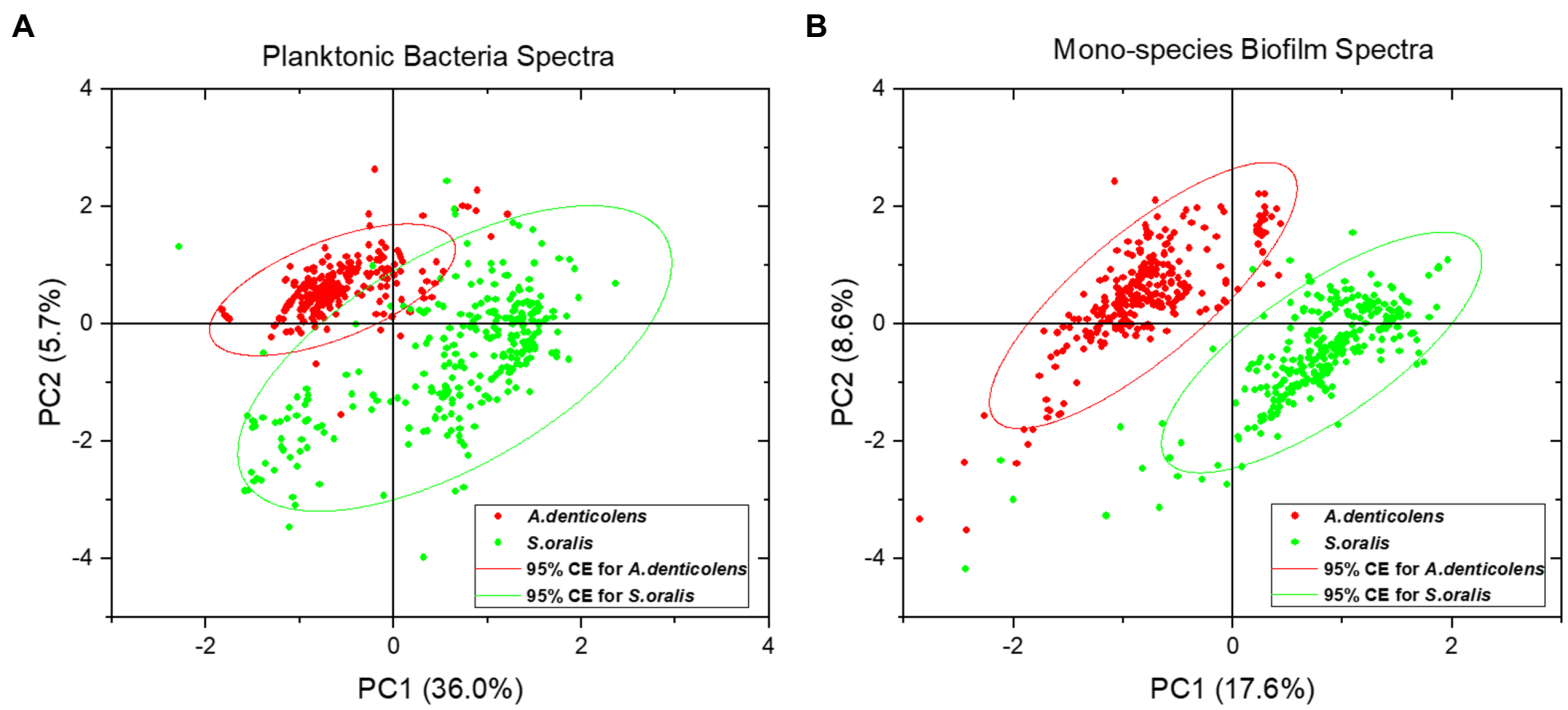
Principle component analysis (PCA) of selected oral bacteria show the distribution of the second-order derivative of spectra (300 spectral samples for each strain) in a score plot. *A. denticolens* is marked in red, while *S. oralis* is marked in green. **(A)** Score plot for planktonic bacteria spectra with a 95% confidence ellipse. **(B)** Score plot for mono-species biofilm spectra with a 95% confidence ellipse.

For data classification and calibration, PCA analysis was performed on the processed Raman data for both planktonic bacteria and mono-species biofilms. Figure 4 shows the score plots of the first two principal components (PC1 and PC2). From the visual inspection of the score plots, it was concluded that the chemometric profile of *A. denticolens* and *S. oralis* could be used to differentiate the two species in both conditions. For planktonic bacteria spectra, the two PCs explained 41.7% of the overall variance present in the dataset (Figure 4A) while for mono-species biofilms, 26.2% of variance was explained (Figure 4B). The PCA analysis demonstrated that two distinguishing clusters were shown, allowing the use of *A. denticolens* and *S. oralis* for the distribution analysis of dual-species biofilms.

Planktonic *S. oralis* showed a bigger variance in the dataset, resulting in a broader spreading of data points within the cluster, while spectra for *A. denticolens* were clustered more narrowly. Nevertheless, a clear difference between the two clusters can be determined (Figure 4A). Even though less variance is described by the two PCs in biofilm conditions, the clusters show a more defined separation of the species. Additionally, both clusters visually show a similar confidence ellipse (CE) size, suggesting that the variance of the spectra within the clusters is comparable (Figure 4B).

### Confirmation of Mapping Analysis Model

Raman distribution images from CA were compared to morphological distribution and FISH distribution. For the morphological differentiation, size and shape of the microorganisms were used as the deciding criteria in ImageJ. Due to the rod shape of *A. denticolens* with a size of 0.2–1.0μm by 2.0–5.0μm and the round cocci shape of *S. oralis* with a size of 0.5–2.0μm, they can be differentiated based on their appearance in a biofilm. Quantification of the area was based on the coverage of each species and was quantified as a ratio. First, FISH confirmed that for the established model, morphology could be used as a simpler visual differentiation between the two species based on their shape. Morphology was used for further analysis as CRM-enabled cross-verification of the species in the absence of staining. Due to technical limitations of the FISH staining procedure, CLSM imaging was not directly compared to other methods unlike Raman and morphology imaging were. Both DBIA samples were analyzed from the same experimental batch but with two samples.

**Figure 5:**
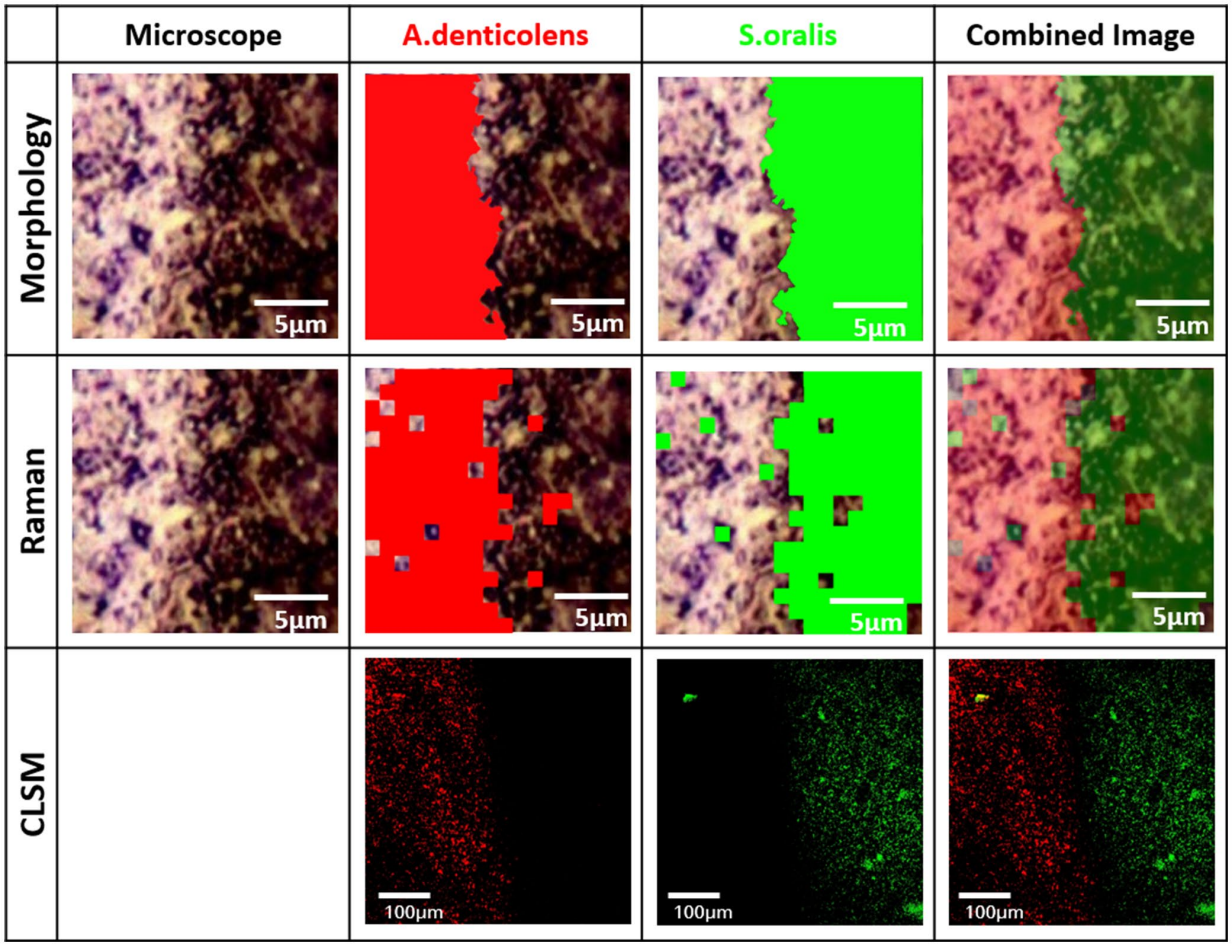
Distribution of *A. denticolens* and *S. oralis* in a dense bacteria interface area (DBIA) sample. Morphological and Raman analysis was performed using the identical random area in a sample. Morphological analysis was done using ImageJ with the plugin MorphoLibJ. Raman analysis was performed using PCA-based cluster analysis with WiRE 5.4. FISH analysis was performed on a new sample from the same experimental batch.

All three images successfully demonstrated two clusters of distribution in the transition area between *A. denticolens* and *S. oralis*. Morphology images showed a defined edge between the two areas, therefore being able to differentiate species more defined based on their bacterial shape (Figure 5). FISH confirmed morphology imaging by indicating the same edge. However, FISH imaging was taken over an image area of 100μm×100μm, while Raman and morphology imaging was done over an image area of 18μm×18μm.

Raman imaging shows a similar edge. Due to the technical limitation of the Raman imaging process (1μm steps), the edge at the transition area is not as specifically assigned, as in morphology imaging. Additionally, some areas that were defined as *A. denticolens* in the morphology image have been classified as *S. oralis* in the Raman images and vice versa. These areas remain minor. Overall, it can be concluded that visually all three images demonstrate a clear edge between the two species. For that reason, CA was confirmed as a method for distribution evaluation of multi-species biofilms using spectral Raman mapping.

### Discrimination of Bacteria in a Two-Species Biofilm

**Figure 6:**
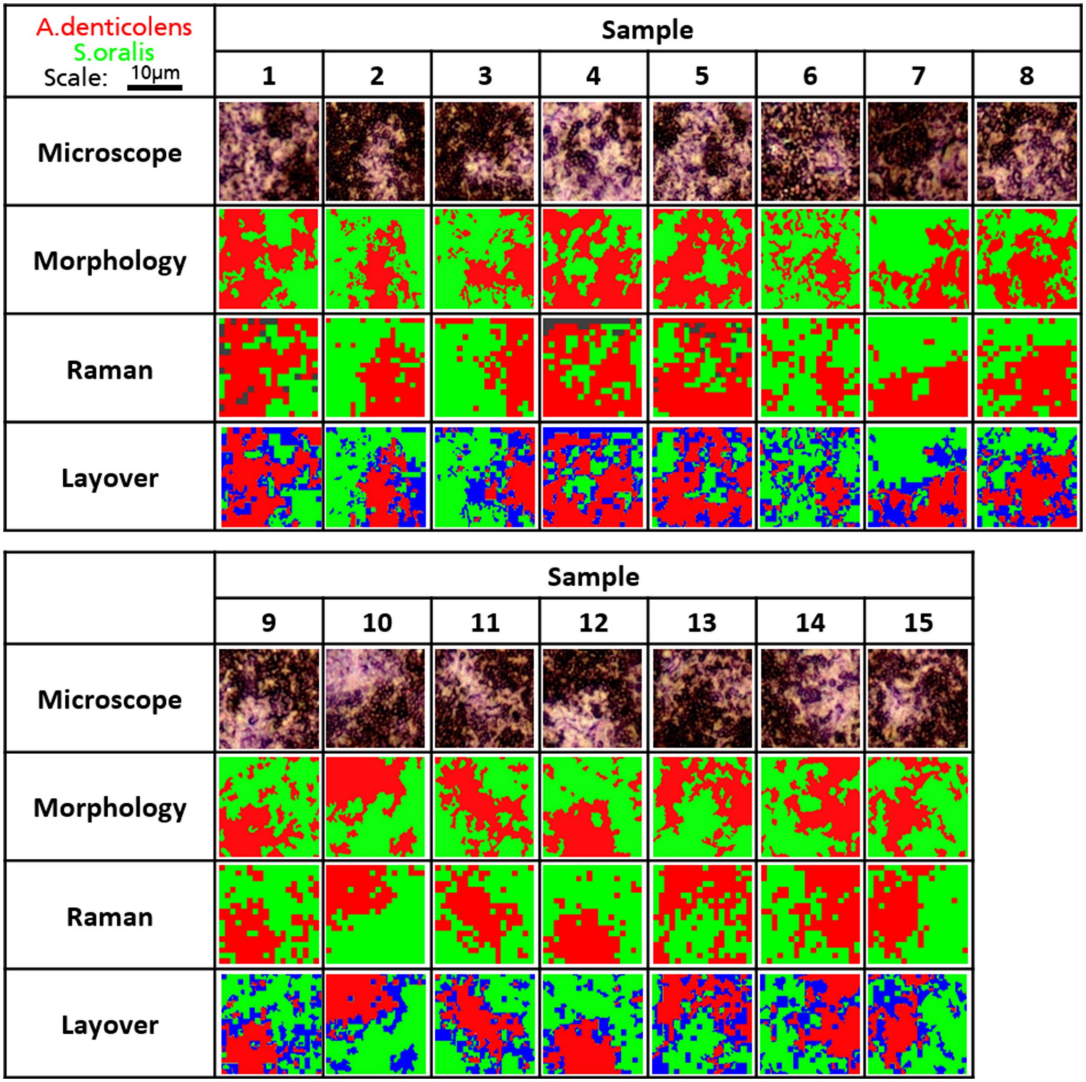
Distribution of *A. denticolens* and *S. oralis* in 15 multi-species biofilms. Morphological and Raman analysis was performed using the identical random area in a sample. Morphological analysis was done using ImageJ with the plugin MorphoLibJ. Raman analysis as performed using PCA-based cluster analysis with WiRE 5.4. Gray areas show unidentified areas. Morphology and Raman images were laid over to show areas that were not classified as the same species and were labeled blue.

The clustering of *A. denticolens* and *S. oralis* in dual-species biofilms is shown in Figure 3. For the analysis and validation of the use of Raman distribution mapping, 15 random areas (18μm×18μm) of biofilm-clustered bacteria were selected and analyzed based on their morphology and Raman spectra in combination with *CA.* Results of the analysis are shown in Figure 6. For Raman analysis, some areas were not able to be classified and are shown in a dark gray color (Samples 1,4,5). After CA, centroid spectra were compared to the calibration spectra (Figures 1B,E) and were colored according to its cluster belonging.

Both cluster images were then compared in layered images (Figure 6). Areas that were not classified by the same bacteria were labeled in blue due to the overlapping of both colors from the morphology and Raman analysis. When looking at the layered images, all major clusters have been identified correctly in all analyzed samples. Small-sized clusters were not detected by Raman analysis. Additionally, differences can be seen in the transition areas between the two clusters where blue areas were most present.

**Figure 7:**
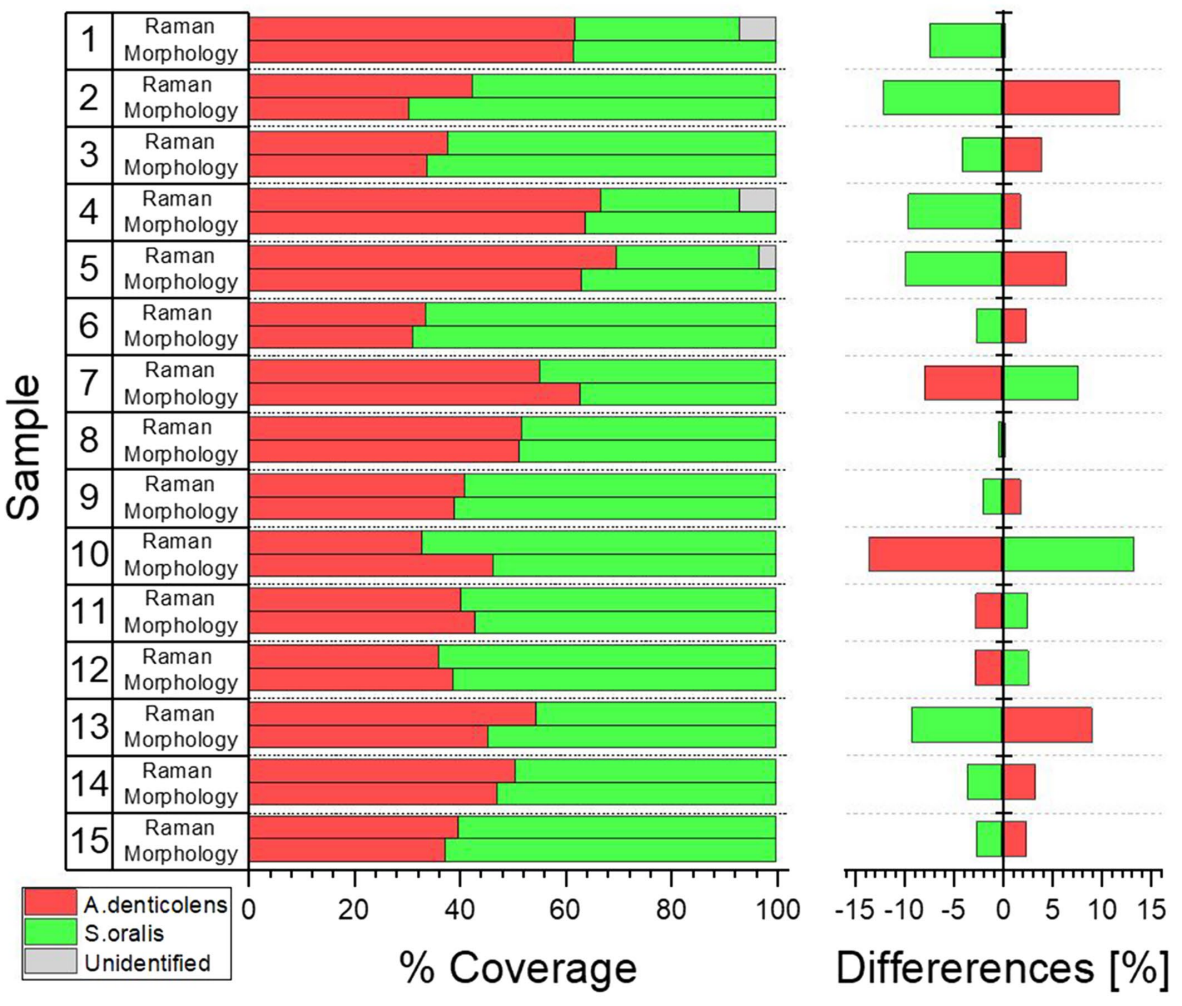
Coverage distribution was calculated from samples shown in Figure 6 for morphology and Raman analysis using ImageJ. Percent difference between the two analysis methods was compared considering morphology analysis as the true distribution.

When looking at the coverage of the two bacteria in the selected areas, they agree well with each other. Differences in coverage between morphology and Raman analysis range between 0.35% (Sample 8) and 13.4% (Sample 10; Figure 7). Samples 1, 4, and 5 with unidentified areas show a reduced coverage of *S. oralis* of 7.0, 7.5, and 3.2%, respectively. There is no trend whether *A. denticolens* or *S. oralis* has been over-identified in Raman samples compared to morphological analysis. Eleven samples showed an over-identification of *A. denticolens*, while four samples showed an over-identification of *S. oralis* when compared to morphology analysis.

This confirmed that Raman analysis was able to determine similar coverages in a two-species biofilm in comparison with morphology. Blue areas in Figure 6 were classified in almost equal amounts as *A. denticolens* (when classified as *S. oralis* by morphology) and *S. oralis* (when classified as *A. denticolens* by morphology). This was further confirmed by the only small percentage differences in the total coverage between the two analysis methods.

## Discussion

In oral biofilm research, CLSM is considered the state-of-the-art technique to analyze the architecture of multi-species biofilms (Abdullah et al., 2019). In contrast, CRM serves as an alternative technique with specific and unique advantages, such as being a non-destructive, chemometric-based method, making it possible to analyze the architecture of biofilms in a vital form. Beier et al. (2010) was able to demonstrate that differentiation of bacteria in a biofilm is possible using staining, while we were able to determine differentiation and mapping based on multivariate analysis as a non-staining method. Additionally, our research underlines the possibility to use Raman microscopy for identification of microorganisms (Pätzold et al., 2006; Stöckel et al., 2016). Our results also suggest that a full bacterial spectrum can be used for the differentiation of species and does not need to be limited to the selection of specific bands (Gieroba et al., 2020; Horiue et al., 2020). This allows for a more stable differentiation method, because multiple bands are considered in our method. Other techniques besides CLSM and CRM have previously been used such as nuclear magnetic resonance (NMR; McLean et al., 2008) that also allows non-destructive measurements on hydroxyapatite surfaces and may be able to differentiate species in a multi-species biofilm based on generated spectra and magnetic resonance imaging and generates similar mappings comparable to CRM based on spectral patterns.

In this study, we developed CRM methods that are able to visualize the distribution of bacterial species in a two-species biofilm model. Using CRM, we could establish the spatial discrimination between native species in cultivated dual-species biofilms. CA and PCA, as the multivariate statistical tools, were able to use chemometric information from Raman spectra to distinguish *A. denticolens* and *S. oralis* in a dual-species biofilm. Mapping biofilms by Raman was further confirmed through a morphology analysis of the same area and CLSM combined with FISH.

By comparing the information of planktonic and biofilm spectra of each species analyzed, it was concluded that they both show very similar chemometric profiles. The biggest differences appear in the height of different bands. These differences can be explained by metabolic changes and up or downregulation of processes when bacteria are forming a biofilm (Svensäter et al., 2001; Marsh, 2004; Wan et al., 2018). Additionally, the formation of extracellular polymeric substances in the cultivation of biofilms may aid to the change in the chemometric signals in the spectra (Sandt et al., 2007). Nevertheless, it was possible to demonstrate that PCA is able to differentiate the selected species in a planktonic and biofilm setup. PCA for planktonic species showed an overlap of the two clusters. While *A. denticolens* showed a dense clustering of the spectra, *S. oralis* was spread broader suggesting an increased variance within the dataset, causing the overlap of clusters. Even though sample preparation for analysis remained consistent throughout the experiments, bacteria that were analyzed at random spots were at different metabolic phases. While the phases may not have a visual influence on the chemometric profile for *A. denticolens*, they may be significant enough for *S. oralis* to show in the variance of the analysis (Strola et al., 2014), resulting in a broader range in the score plot (Figure 4). For that reason, calibration dataset differentiation may improve by increasing the number of spectra in the dataset to better represent the complete data distribution, which then leads to more accurate predictability of species (Guo, 2018). It also needs to be noted that as part of the sample preparation, bacteria were washed with DI water to keep the sample preparation consistent with biofilm sample preparation. However, DI water may damage the bacterial cell, releasing substances from the cell which could lead insufficient measurements. The effect of rinsing samples with DI water on the Raman “fingerprint region” need to be evaluated in future research. Physiological solutions such as PBS were not considered because it showed a negative effect on peak intensities of bacteria using SERS (Alula et al., 2017). Nevertheless, the multivariate analysis of biofilm spectra showed two defined clusters with only minor outliers, which suggests less variance within the cluster and thus allows for a more specific analysis model compared to planktonic species. The analysis demonstrated sufficient evidence of differentiation using PCA for data sampling from multiple samples and CA for mapping of biofilms.

The analysis of DBIA samples was used to confirm the use of CA for the analysis of dual-species biofilms. We were able to show that cluster mapping based on Raman spectra was comparable with the morphology analysis and was able to determine two areas of predominantly one species. While morphology showed two distinct areas, CA also classified areas with the other species not detected by morphology (Figure 6). One reason can be that the morphology analysis was not able to detect the species correctly. The more likely explanation, however, is that because the overlap of clusters present in the PCA analysis, the CA model was also not as species-specific, thus causing misclassification (Ben-Hur and Guyon, 2003). Overall, CA coupled with Raman analysis confirmed that CA could be used as a method to differentiate species in a DBIA samples because differences between morphology and Raman analysis remain only minor.

For dual-species biofilm models, CA was able to successfully map species of a specific area in focus. Additionally, determinations of coverages between morphology and Raman mapping were comparable, indicating that similar amounts of *S. oralis* and *A.denticolens* were detected in selected areas. This confirms that the differences in the chemometric profile are significant enough to allow the mapping of the two species in a biofilm model. Nevertheless, the Raman analysis showed some limitations mostly visible in the transition areas of clusters where bacteria were classified differently than when compared to morphology. An explanation of different classifications can be that signals from layers below the focused layer were not the same species (Ramirez-Mora et al., 2019; Gieroba et al., 2020). This effect causes the detection of both species and generates a mixed chemometric profile of both species, where the model then classified spectra into a different cluster. When comparing these areas to CLSM images, yellow areas in the transition areas confirm both species to be present (Figure 3). This suggests that indeed the Raman spectra in the transition areas carry chemometric information from both species that results in arbitrary classifications.

In future research, it will be pivotal to determine the limits of this analysis technique. One downfall of this technique is the technical limitation of the analysis by only receiving spectral information in 1μm steps. This limitation results in the lack of being able to detect small sized clusters. Additionally, the used acquisition method is substance for optimization. Spectral acquisitions of areas required around 60min for an area of 324μm^2^, which is the result of numbers of accumulation readings per point and long exposure times of every acquisition. It will be important to determine how far accumulations and exposure times can be reduced while still being able to receive differentiation of species in a multi-species biofilm and keep the same resolution. Furthermore, the technique can be improved by reducing laser power in order to minimize the possible artifact from chemometric spectral information from layers below the focused layer. While CRM can be used as a non-destructive technique, high laser power and large area mapping may result in the burning the biofilm and the consequential destruction of the sample and structure. This needs to be considered in order to use optimized acquisition settings to ensure the non-destructive nature of this technology.

In conclusion, the described technique was able to differentiate two species in a multi-species biofilm model. Next steps of establishing Raman microscopy for biofilm mapping are: (1) the analysis of more than two-species in a biofilm and (2) considering the analysis of three-dimensional views. To our knowledge, this research laid the groundwork for the use of Raman microscopy in combination with multivariate analysis techniques to map multi-species biofilms for the first time. Nonetheless, questions remain unanswered in order to make it a competitive technique to CLSM. With the results of this study, it is possible to establish Raman mapping as a complementary technique to CLSM because it is able to give information on the chemometrics of species in biofilms. With the advancements of the technique in the future, it may be possible to use Raman as an alternative and synergistic method for biofilm mapping.

## Data Availability Statement

The raw data supporting the conclusions of this article will be made available by the authors, without undue reservation.

## Author Contributions

LK contributed to conceptualization, analysis, methodology, software, validation, visualization, and writing and revising the manuscript. KW contributed to conceptualization, supervision, interpretation, and drafting and revising the manuscript. RC-V contributed to methodology, supervision, and drafting and revising the manuscript. SR contributed to conceptualization, project administration, supervision, and drafting and revising the manuscript. All authors contributed to the article and approved the submitted version.

## Funding

This work was funded by the European Union’s Horizon 2020 Research Innovation Program under grant agreement no. 722871 in the scope of the Marie Skłodowska-Curie Action ITN BioClean to LK. P&G and other partners contributed time, industrial supervision, training and technical guidance or expertise, access to equipment, and placements in industry, and coauthored and reviewed associated papers, including this submission all in line with EU Horizon 2020 funding rules. More details can be found on the Horizon 2020 website (http://www.biocleanh2020.eu/index.php). The funders had no role in study design, data collection and analysis, or decision to publish the manuscript.

## Conflict of Interest

The authors declare that the research was conducted in the absence of any commercial or financial relationships that could be construed as a potential conflict of interest.

## Publisher’s Note

All claims expressed in this article are solely those of the authors and do not necessarily represent those of their affiliated organizations, or those of the publisher, the editors and the reviewers. Any product that may be evaluated in this article, or claim that may be made by its manufacturer, is not guaranteed or endorsed by the publisher.

## Supplementary Material

Supplementary Figure 1 Individual Z-stack CLSM images of an in-vitro subgingival biofilm model. Z-steps size of 1.018 µm was used with specific 16S rRNA FISH probes of *S. oralis* (MIT447, green) and *A. denticolens* (ACT476, red). Magnification of 100x was used.
