# Supplementary figures and images for: Mapping of a Subgingival Dual-Species Biofilm Model Using Confocal Raman Microscopy
Source: Front Microbiol. 2021 Oct 5;12:729720. doi: 10.3389/fmicb.2021.729720 (PMC8525910; doi:10.3389/fmicb.2021.729720)

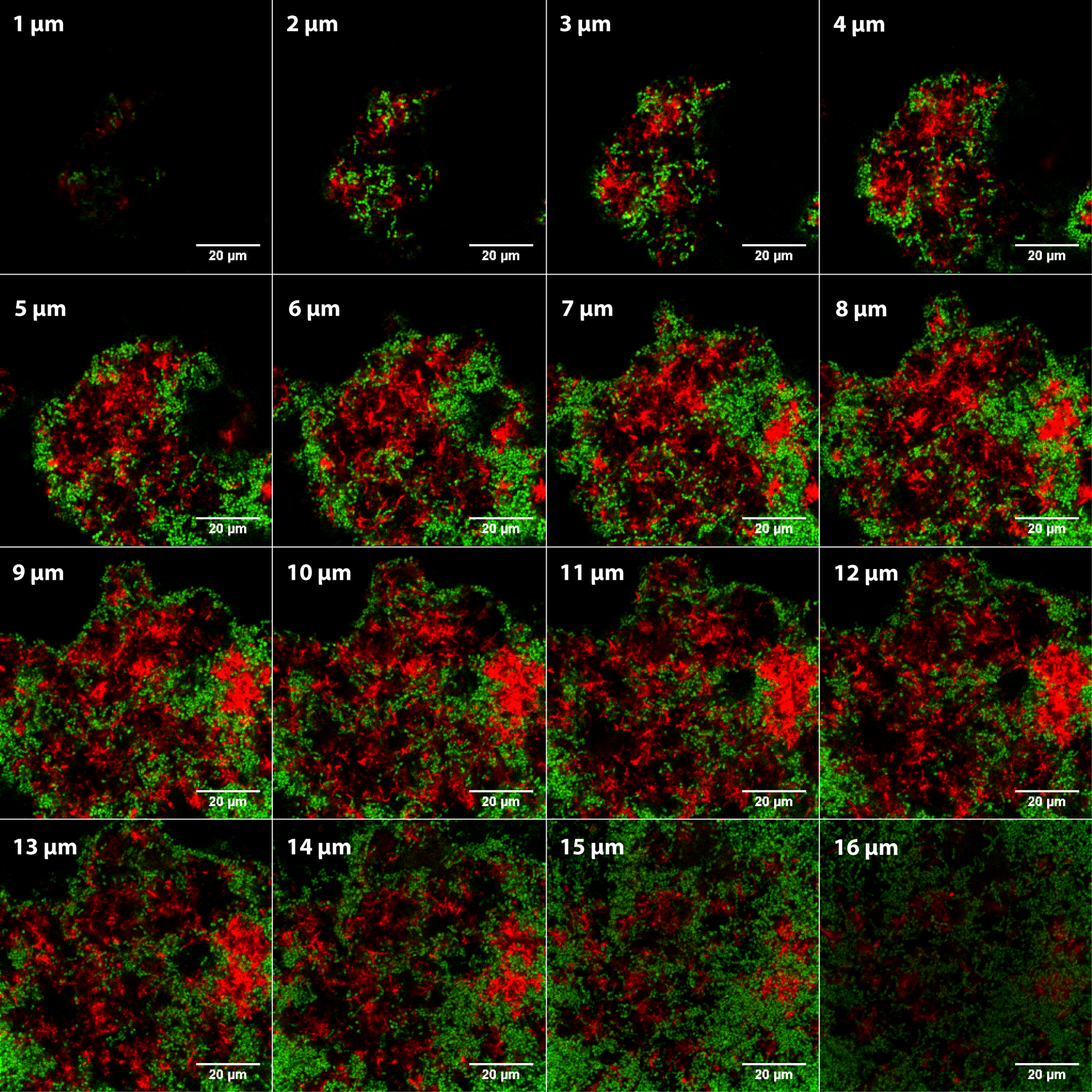

Supplement: Supplementary Figure 1 — Individual Z-stack CLSM images of an in-vitro subgingival biofilm model. Z-steps size of 1.018 µm was used with specific 16S rRNA FISH probes of S. oralis (MIT447, green) and A. denticolens (ACT476, red). Magnification of 100x was used. [file Image_1.TIF]
